# Identifying novel proteins underlying schizophrenia via integrating pQTLs of the plasma, CSF, and brain with GWAS summary data

**DOI:** 10.1186/s12916-022-02679-5

**Published:** 2022-12-08

**Authors:** Xiaojing Gu, Meng Dou, Weiming Su, Zheng Jiang, Qingqing Duan, Bei Cao, Yongping Chen

**Affiliations:** 1 Mental Health Center, West China Hospital, Sichuan University, Chengdu, 610041 Sichuan China; 2 Chengdu Institute of Computer Application, Chinese Academy of Sciences, Chengdu, 610041 Sichuan China; 3 Department of Neurology, West China Hospital, Sichuan University, Chengdu, 610041 Sichuan China; 4 Lab of Neurodegenerative Disorders, Clinical Institute of Inflammation and Immunology (CIII), Frontiers Science Center for Disease-Related Molecular Network, West China Hospital, Sichuan University, Chengdu, 610041 Sichuan China; 5 Centre for Rare Diseases, West China Hospital, Sichuan University, Chengdu, 610041 Sichuan China

**Keywords:** Protein quantitative trait loci, Schizophrenia, Mendelian randomization, Complement 4, Neuroinflammation

## Abstract

**Background:**

Schizophrenia (SCZ) is a chronic and severe mental illness with no cure so far. Mendelian randomization (MR) is a genetic method widely used to explore etiologies of complex traits. In the current study, we aimed to identify novel proteins underlying SCZ with a systematic analytical approach.

**Methods:**

We integrated protein quantitative trait loci (pQTLs) of the brain, cerebrospinal fluid (CSF), and plasma with the latest and largest SCZ genome-wide association study (GWAS) via a systematic analytical framework, including two-sample MR analysis, Steiger filtering analysis, and Bayesian colocalization analysis.

**Results:**

The genetically determined protein level of C4A/C4B (OR = 0.70, *p* = 1.66E−07) in the brain and ACP5 (OR = 0.42, *p* = 3.73E−05), CNTN2 (OR = 0.62, *p* = 2.57E−04), and PLA2G7 (OR = 0.71, *p* = 1.48E−04) in the CSF was associated with a lower risk of SCZ, while the genetically determined protein level of TIE1 (OR = 3.46, *p* = 4.76E−05), BCL6 (OR = 3.63, *p* = 1.59E−07), and MICB (OR = 4.49, *p* = 2.31E−11) in the CSF were associated with an increased risk for SCZ. Pathway enrichment analysis indicated that genetically determined proteins suggestively associated with SCZ were enriched in the biological process of the immune response.

**Conclusion:**

In conclusion, we identified one protein in the brain and six proteins in the CSF that showed supporting evidence of being potentially associated with SCZ, which could provide insights into future mechanistic studies to find new treatments for the disease. Our results also supported the important role of neuroinflammation in the pathogenesis of SCZ.

**Supplementary Information:**

The online version contains supplementary material available at 10.1186/s12916-022-02679-5.

## Background

Schizophrenia (SCZ) is a chronic, severe mental illness with a global age-standardized point prevalence estimated to be 0.28%, which loads a great burden on patients’ families and society [1]. Patients with SCZ can present with positive symptoms (such as delusions and hallucinations), negative symptoms (including lack of motivation and social withdrawal), and cognitive symptoms (such as deficits in working memory, executive function, and processing speed) [2]. Although tremendous efforts have been put into exploring effective therapeutics for SCZ, there is no cure so far [3], which may be caused by the insufficient understanding of its etiology.

The etiology of SCZ is complex, and genetic factors have been proven to play an important role in the pathogenesis of SCZ. On the one hand, previous twin studies have consistently shown a large genetic component to SCZ, with heritability estimated at around 80% [4]. On the other hand, based on the common disease-common variant hypothesis, genome-wide association studies (GWAS) have been applied as an unbiased, data-driven approach to identifying several loci associated with SCZ [5–8]. However, the GWAS design cannot reliably pinpoint potentially causal genes for explaining the high heritability of SCZ.

Mendelian randomization (MR) is a genetic method using the genome-wide significant single nucleotide polymorphisms (SNPs) that were strongly associated with the exposure as instrumental variables (IVs), to investigate the causal link between exposure and outcome [9]. It has been widely used in exploring etiologies of complex diseases. Likewise, supposing that we choose the SNPs associated with the abundance of a protein (protein quantitative trait loci (pQTLs)) as exposure and use the SCZ as the outcome, we can infer the potential causal link between the proteins on SCZ. This will help to identify novel proteins underlying SCZ, uncover disease pathogenesis, and guide therapeutics development [10, 11].

Therefore, in the current study, we combined pQTLs from neurologically relevant tissues (brain, CSF) and plasma [12] and used the two-sample MR method, Steiger filtering analysis, and Bayesian colocalization analysis to explore the relationship between genetic-determined protein levels and SCZ. Then, we also correlated the MR effects from the brain, CSF, and plasma and further analyzed protein-protein interaction (PPI) between SCZ-related proteins. This integration will help to identify tissue-shared and tissue-specific proteins that play important roles in SCZ, which will help in future mechanistic study and drug discovery.

## Methods

**Fig. 1:**
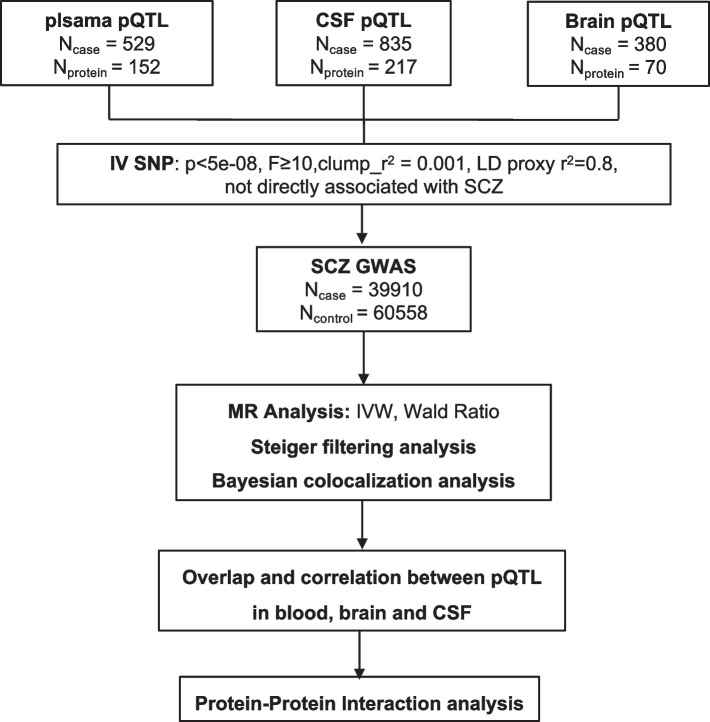
Flowchart of the study. We integrated pQTL data derived from brain, CSF, and plasma and SCZ GWAS datasets via MR, Steiger filtering analysis, and Bayesian colocalization analysis. Then, we compared the consistency and differences among the three different tissues and further mapped the interaction networks between the identified proteins

The flowchart of the study was presented in Fig. 1.

### Datasets

The information about the datasets used in the current study was listed in Additional file 1: Table S1. Participants were all of European ancestry.

#### pQTL dataset

We obtained the pQTLs of the brain, CSF, and plasma from a recent study [12], and the demographic characteristics of the participants were listed in Additional file 1: Table S2. Briefly, participants with Alzheimer’s disease (AD) and healthy controls of European ancestry were included to measure the abundance of 1305 proteins using an aptamer-based platform in CSF (*n* = 835), plasma (*n* = 529), and brain (parietal lobes) (*n* = 380) samples. Then, to identify the association between genotype and protein levels within each tissue, genome-wide association analyses of 14.06 million imputed autosomal common variants (minor allele frequency ≥ 0.02) were performed against protein levels in each tissue. Moreover, because the study included cognitively normal older adults and patients with AD, additional analyses were performed to determine if any of the pQTLs were disease or age-specific, and the results showed that associations of the genetic variants with protein levels were not disease-specific, and most of the pQTLs were not age-specific. The detailed methods can be found in the original study [12]. In general, we selected pQTLs that passed the stringent genome-wide threshold of *p* < 5E−08 as IVs.

#### SCZ dataset

The summary statistics of SCZ were obtained from the latest SCZ GWAS conducted by the SCZ Working Group of the Psychiatric Genomics Consortium (PGC), which included 39,910 SCZ cases and 60,558 controls of European ancestry [8]. Written consent was obtained from all individuals enrolled in this study, and the study was approved by the institutional review board described in the original study [5, 8].

### Two-sample Mendelian randomization

MR analysis utilizes SNPs as IVs to explore the causal effects of defined exposure on an outcome, which has been widely applied in identifying the genetic etiology of complex illnesses through integrating the quantitative trait loci data [13, 14]. In this study, we used the pQTL datasets as the exposure [12] and SCZ GWAS [5, 8] as the outcome to perform MR.

#### IV selection

The most important and fundamental step of MR is to include eligible IVs. To identify eligible IVs, three key assumptions must be met [9]. Assumption 1 (relevance assumption) refers to that the genetic variant should be directly associated with the exposure. Therefore, to meet assumption 1, on the one hand, we restricted the SNPs to be directly associated with the exposure at the *p*-value < 5E−08 (genome-wide significant threshold); on the other hand, we selected robust SNPs judged by *F*-statistics ≥ 10 [15] (Additional file 1: Table S3). Assumption 2 (independence assumption) is that the genetic variant should not be directly related to confounding factors, which can be calculated as horizontal pleiotropy in the post-MR analysis. Assumption 3 (exclusion assumption) is that the genetic variant should not be directly associated with the outcome. To meet this assumption, the PhenoScanner database was searched for each SNP to see whether they were significantly associated with the outcome (*p* < 5E−08) [16] (Additional file 1: Table S4), and those SNPs directly associated with the outcome were not included.

#### Mendelian randomization

Once eligible IVs were selected, they were linkage disequilibrium (LD) clumped with *r*^2^ < 0.001 in 10 megabase distance. After clumping, most of the pQTLs have at most 2 eligible IVs. Next, the IVs were extracted from the outcome trait and were harmonized in both exposure and outcome GWAS. In this step, palindromic SNPs with intermediate allele frequency were removed. Moreover, if a particular requested SNP is not present in the outcome GWAS, then an SNP (proxy) that is in LD with the requested SNP (target) will be searched, which was defined using 1000 genomes European sample data (*r*^2^ ≥ 0.8). Once the exposure and outcome data are harmonized, MR can be performed. Because most of the pQTLs have at most 2 eligible IVs, under this condition, only the Wald ratio method (1 IV SNP) and inverse-variance-weighted (IVW) method (2 IV SNPs) can be calculated according to the default settings in the MR package, while other sensitivity analyses including MR-Egger, weighted mode, and weighted median mode cannot be performed. Moreover, because of the limited number of IVs, post-MR analyses such as Cochran’s *Q* test for heterogeneity, MR-Egger intercept test for horizontal pleiotropy, MRPRESSO test for the outlier, and *I*^2^GX test for “no measurement error” were not able to be performed [9]. As a result, the suggested threshold of the *p*-value (*p* < 0.05) and Bonferroni correction thresholds (*p* < 0.05/number of proteins analyzed) were used to prioritize proteins for further follow-up study, and to ensure the causation was not distorted by the presence of reverse causation, the Steiger filtering method was applied [17]. *p* < 0.05 indicated that the effect direction is from exposure to an outcome. The steps above were implemented using the “TwoSampleMR” R package (github.com/MRCIEU/TwoSampleMR) [7].

### Bayesian colocalization analysis

To avoid the signals discovered by MR that might arise from linkage disequilibrium or pleiotropy, Bayesian colocalization analysis was performed to assess whether these two association signals (pQTLs and SCZ GWAS) were consistent with a shared causal variant [18]. The analysis was conducted with COLOC (https://rdrr.io/cran/coloc/) in the R package with default parameters, which tested the posterior probability of 5 hypotheses: H_0_: no association with either trait; H_1_: association with trait 1 (pQTL), not with trait 2 (SCZ GWAS); H_2_: association with trait 2 (SCZ GWAS), not with trait 1 (pQTL); H_3_: association with trait 1 (pQTL) and trait 2 (SCZ GWAS), two independent SNPs; H_4_: association with trait 1 (pQTL) and trait 2 (SCZ GWAS), one shared SNP [18], and a posterior probability of hypothesis 4 (PPH_4_) > 0.8 was considered as that the two association signals are consistent with a shared causal variant [18].

### Pearson correlation of MR effects

We wondered whether there would be correlations between the brain, CSF, and plasma-identified QTLs. Therefore, we investigated the correlation between the shared QTLs identified in the brain, CSF, and plasma using effect estimates (beta) from the MR analysis by Pearson correlation analysis. Since the amount of pQTLs was relatively small, let alone the overlapping proteins, we first set no threshold for pQTLs to ensure enough shared pQTLs in the correlation analysis. Next, a *p*-threshold at 0.05 was also applied to ensure a stringent correlation.

### Protein-protein network

To explore the underlying pathways of the significant proteins, we investigated whether there were PPI networks and enrichment of pathways between these proteins that survived multiple testing. Moreover, to gain more information, we also performed PPI among those proteins with *p* < 0.05. PPI and Gene Ontology (GO) enrichment were found in the Search Tool for the Retrieval of Interacting Genes (STRING) database version 11.5 (https://string-DB.org/) [19].

## Results

### Associations between genetically predicted protein levels in the brain with SCZ

**Fig. 2:**
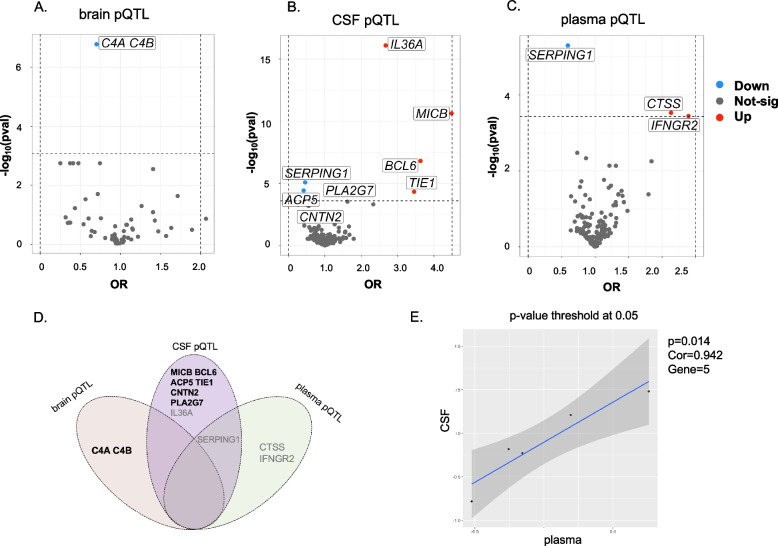
MR results for pQTLs and SCZ. **A** Brain pQTL and SCZ. **B** CSF pQTL and SCZ. **C** Plasma pQTL and SCZ. The dots colored in blue or red represent the genes that passed the Bonferroni-corrected *p*-value thresholds (*P* < 0.05/number of analyzed genes). **D** Venn plot of the top significant proteins shared by brain, CSF, and plasma pQTLs; the boldly labeled genes indicated those have evidence of colocalization. **E** Correlation of MR effect between the plasma and CSF (*P*-value threshold < 0.05)

The MR analysis of brain pQTLs only identified one genetically determined protein level to be associated with the risk for SCZ after multiple testing corrections (*p* < 8.3E−04 [0.05/6]) (Table 1, Fig. 2A, and Additional file 1: Table S5). Specifically, one standard deviation increase of the complement 4A and 4B (C4A/C4B) protein level in the brain was associated with an approximately 30% decreased risk of developing SCZ (OR = 0.70, *p* = 1.66E−07). Steiger filtering analysis supported that C4A/C4B had correct causal direction from protein level to the development of SCZ, and Bayesian colocalization analysis confirmed that the pQTL and SCZ were mediated by the shared variant (PPH_4_ = 0.969), which further provided supporting evidence for its potentially causal association with SCZ (Table 1).

**Table 1:** Candidate proteins showing supporting evidence of potential causality for schizophrenia by MR analysis, Steiger filtering analysis, and Bayesian colocalization analysis

Tissue	Protein	MR analysis					Steiger filtering analysis		Bayesian colocalization analysis		Evidence of causal association
		Method	nsnp	pval	Beta	OR (95%CI)	pval	Causal direction	PPH4	Colocalization	
Brain	C4A C4B	Wald ratio	1	1.66E−07	− 0.35	0.70 (0.62–0.80)	1.29E−15	Correct	0.969	Yes	Yes
CSF	PLA2G7	Wald ratio	1	1.48E−04	− 0.35	0.71 (0.59–0.85)	7.41E−63	Correct	0.870	Yes	Yes
CSF	IL36A	Wald ratio	1	8.07E−17	0.98	2.67 (2.12–3.16)	1.08E−73	Correct	1.92E−23	No	No
CSF	TIE1	Wald ratio	1	4.76E−05	1.24	3.46 (1.90–6.28)	1.72E−07	Correct	0.942	Yes	Yes
CSF	BCL6	Wald ratio	1	1.59E−07	1.29	3.63 (2.24–5.58)	1.60E−11	Correct	1.000	Yes	Yes
CSF	MICB	Wald ratio	1	2.31E−11	1.50	4.49 (2.89–6.97)	8.06E−55	Correct	0.999	Yes	Yes
CSF	ACP5	Wald ratio	1	3.73E−05	− 0.87	0.42 (0.28–0.63)	2.13E−17	Correct	0.959	Yes	Yes
CSF	SERPING1	Wald ratio	1	8.25E−06	− 0.78	0.46 (0.33–0.65)	4.76E−20	Correct	0.090	No	No
CSF	CNTN2	Wald ratio	1	2.57E−04	− 0.47	0.62 (0.49–0.80)	4.86E−47	Correct	0.821	Yes	Yes
Plasma	CTSS	Wald ratio	1	2.94E−04	0.76	2.13 (1.42–3.22)	4.73E−27	Correct	0.110	No	No
Plasma	IFNGR2	Wald ratio	1	3.59E−04	0.87	2.40 (148–3.87)	3.22E−08	Correct	0.747	No	No
Plasma	SERPING1	Wald ratio	1	5.07E−06	− 0.52	0.59 (0.47–0.74)	1.77E−15	Correct	0.650	No	No

### Associations between genetically predicted protein levels in CSF with SCZ

After multiple testing corrections (*p* < 2.63E−04 [0.05/190]), the MR analysis identified 8 genetically predicted proteins in the CSF to be associated with SCZ (Table 1, Fig. 2B, and Additional file 1: Table S6). Specifically, the increased protein abundance of 4 proteins was significantly associated with an increased risk of SCZ, including interleukin 36A (IL36A, OR = 2.67, *p* = 8.07E−17), tyrosine kinase with immuno-globulin-like and EGF-like domains 1 (TIE1, OR = 3.46, *p* = 4.76E−05), BCL6 transcription repressor (BCL6, OR = 3.63, *p* = 1.59E−07), and MHC class I polypeptide-related sequence B (MICB, OR = 4.49, *p* = 2.31E−11), while the increased protein abundance of the remaining 4 proteins was significantly associated with a decreased risk of SCZ, including acid phosphatase 5 (ACP5, OR = 0.42, *p* = 3.73E−05), protease inhibitor C1 (SERPING1, OR = 0.46, *p* = 8.25E−06), contactin 2 (CNTN2, OR = 0.62, *p* = 2.57E−04), and phospholipase A2 group VII (PLA2G7, OR = 0.71, *p* = 1.48E−04). The Steiger filtering analysis indicated that all MR-identified proteins had the correct causal direction from protein level to SCZ. However, in the colocalization analysis, only 6 proteins showed evidence for genetic colocalization with SCZ, including TIE1, BCL6, MICB, ACP5, CNTN2, and PLA2G7, which further provided supporting evidence for their potential causal association with SCZ (Table 1).

### Associations between genetically predicted protein levels in plasma with SCZ

After multiple testing corrections (*p* < 3.81E−04 [0.05/131]), the MR analysis identified three genetically predicted plasma proteins to be associated with SCZ (Table 1, Fig. 2C, and Additional file 1: Table S7). Specifically, the increased protein abundance of two proteins was associated with an increased risk of SCZ, including cathepsin S (CTSS, OR = 2.13, *p* = 2.94E−04) and interferon gamma receptor 2 (IFNGR2, OR = 2.40, *p* = 3.59E−04). In contrast, the increased abundance of SERPING1 (OR = 0.59, *p* = 5.07E−06) was significantly associated with a decreased risk of SCZ. Although Steiger filtering indicated correct causal direction from the protein level to the development of SCZ, the Bayesian colocalization analysis indicated that none of the proteins had evidence for genetic colocalization (Table 1).

### Summary of MR findings

Our findings provided supporting evidence for a potential causal relationship between C4A/C4B in the brain and TIE1, BCL6, MICB, ACP5, CNTN2, and PLA2G7 in the CSF and SCZ, because MR analysis found the potential causal association, the Steiger filtering test confirmed that the direction was correct, and the Bayesian colocalization analysis further supported that the protein profiles and SCZ were consistent in the shared variants. However, the association of IL36A in CSF, SERPING1 in CSF and plasma, and CTSS and IFNGR2 in plasma were less robust, because although the MR analysis identified the potential causal association, the colocalization analysis failed to find evidence of colocalization, which indicated that the potential causal association discovered by MR might arise from linkage disequilibrium or pleiotropy (Table 1 and Fig. 2D).

### Consistency comparison by correlation analysis

To further explore the correlation between brain-based, CSF-based, and plasma-based proteins, we compared the MR effect estimates of the shared proteins. The MR effects between brain proteins and CSF proteins showed a robust positive correlation (no *p*-value threshold, Pearson correlation = 0.642, *p*-value = 2.84E−07, number of proteins = 52) (Additional file 2: Fig. S1A), and the correlation was strengthened when limited *p*-value threshold at 0.05 (Pearson correlation = 0.972, *p*-value = 0.014, number of proteins = 5) (Fig. 2E). However, the MR effects between plasma proteins and brain proteins (*p*-value = 0.113, number of proteins = 13), and brain proteins with CSF proteins (*p*-value = 0.147, number of proteins = 17) both did not show correlations at no *p*-value threshold (Additional file 2: Fig. S1B-C).

### PPI network

**Fig. 3:**
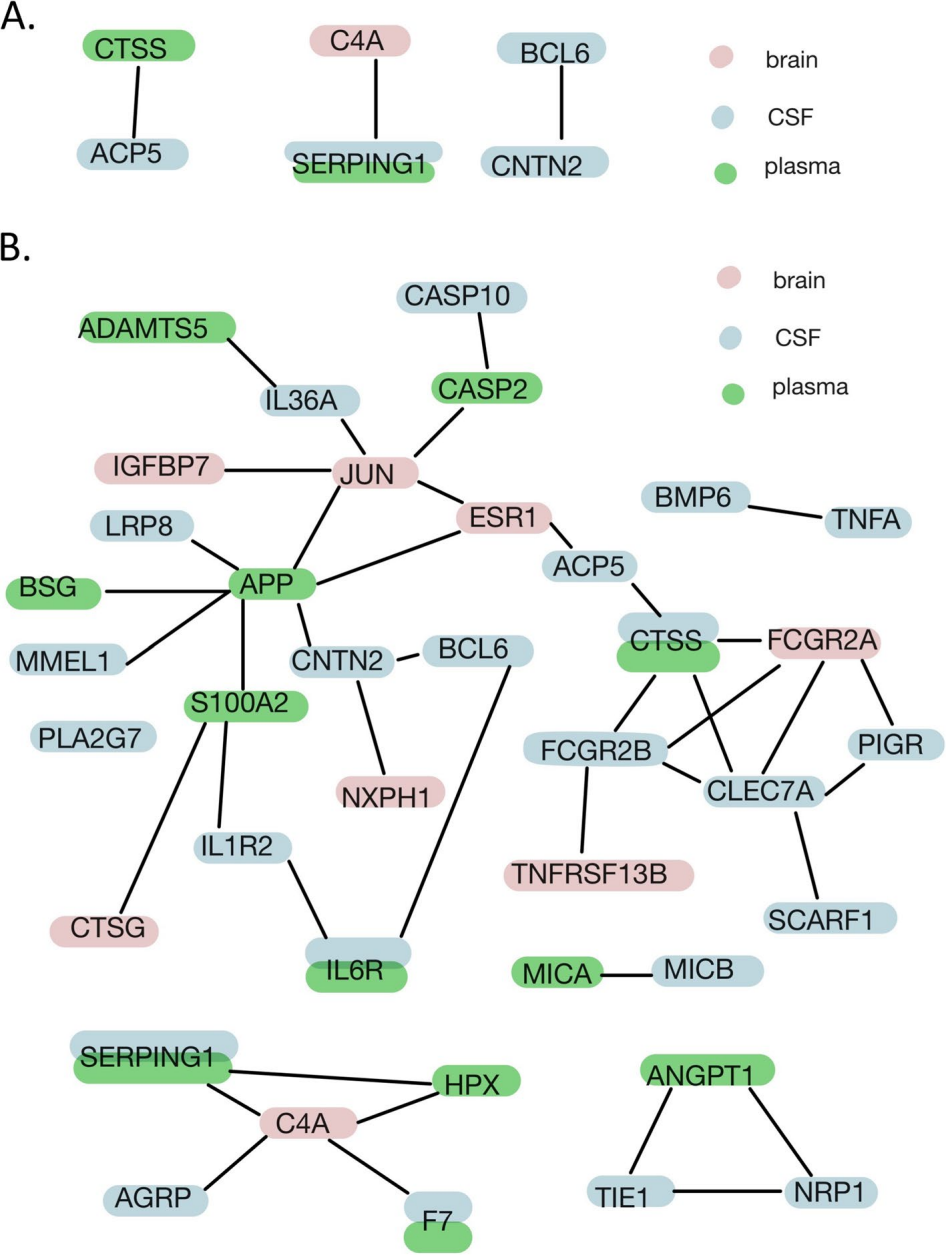
Protein-protein interaction. **A** Protein-protein interaction network between significant schizophrenia-related proteins (*P* passed the multiple testing) from the brain, CSF, and plasma. **B** Protein-protein interaction network between suggestive schizophrenia-related proteins(*P* < 0.05) from the brain, CSF, and plasma. Pink labeled: brain protein; blue labeled: CSF protein; green labeled: plasma protein

We found protein interactions between the significant proteins from multiple tissues. For example, C4A/C4B was associated with SCZ in the brain, SERPING1 was associated with SCZ in the CSF and plasma, and they were found to interact with each other in the PPI network (Fig. 3A). Moreover, using the proteins which were suggestive of being associated with the risk for SCZ (*p* < 0.05) from three tissues (brain, CSF, and plasma) (Additional file 1: Table S5-S7), the PPI networks in STRING revealed that the whole network was significantly enriched (*p* = 4.99E−08) (Fig. 3B), and GO enrichment analysis indicated these suggestive proteins were enriched in the biological process of “regulation of response to external stimulus” and “regulation of immune system process” (Additional file 1: Table S4).

## Discussion

Although significant developments have been made in sequencing methods and bioinformatics tools, the genetic etiology of SCZ remains largely unknown. The current study applied a systematic pipeline combing MR, Steiger filtering analysis, and Bayesian colocalization analysis with the brain-, CSF-, and plasma-based pQTL datasets to identify novel proteins underlying SCZ. We provided supporting evidence for the potential causal association between genetically determined protein levels of C4A/C4B in the brain and TIE1, BCL6, MICB, ACP5, CNTN2, and PLA2G7 in the CSF and SCZ. Subsequently, we found a robust correlation of the MR effects between plasma- and CSF-based proteins. Last but not least, we found that the suggestive proteins were enriched in the biological process of “regulation of response to external stimulus” and “regulation of immune system process.”

Some studies have explored the expression quantitative trait loci (eQTL) and/or pQTL profiles in SCZ patients. For example, a study using brain pQTL and eQTL datasets with proteome-wide association analysis (PWAS) and transcriptome-wide association study (TWAS) revealed 14 genes associated with the risk for SCZ [20]. Another study measured the eQTL and pQTL of the antihypertensive drug target genes in the blood, CSF, and brain with MR methods and found an adverse association of lower ACE messenger RNA and protein levels with SCZ risk [21]. Wang et al. used blood eQTL and GWAS data of SCZ in East Asian populations with TWAS and summary data-based Mendelian randomization (SMR) and revealed TMEM180 as a SCZ risk gene [22]. Baird et al. used the brain eQTL dataset with MR design and found 23 potentially causal genes with evidence of a shared genetic effect between gene expression (eQTL) and SCZ risk [23]. However, limitations in these studies should be noteworthy. Firstly, like GWAS, the design of TWAS and PWAS was only able to identify the association but not pinpoint the causality. Furthermore, pQTL in the blood could not reflect the biological process in the brain because of the blood-brain barrier. Last but not least, eQTL mapping could not fully identify the functional variants and genes driving GWAS signals, because many genetic variants alter protein levels without affecting transcript levels [24]. Therefore, our study has several advantages over previous studies. First, our study applied several independent but complementary methods to identify novel proteins for SCZ, including the MR analysis to discover the potential causal association, the Steiger filtering analysis to ensure the correct direction of the association, and Bayesian colocalization analysis to verify that the potential causal association was not distorted by LD and pleiotropy. Second, we integrated pQTLs from multiple-tissue (brain, CSF, and plasma) to comprehensively explore the crucial proteins involved in the pathogenesis of SCZ. Last but not least, we performed the analysis with the latest and largest SCZ GWAS dataset.

In the current study, we found that C4A/C4B in the brain showed a protective role against SCZ. C4A and C4B are two highly conserved isoforms of C4, which reside in the major histocompatibility complex (MHC) locus [25]. The previous study has shown that alleles increasing C4A expression correlate with increased SCZ risk, but alleles that increase C4B expression do not alter SCZ risk [26], and the plasma level of C4 is increased in patients with SCZ, as well as inversely correlated with the cortex thickness [27, 28]. In vivo studies also showed the overexpression of C4A in mice would reduce cortical synapse density, increase microglial engulfment of synapses, and lead to behavioral changes in the mice [25]. However, our study with MR design found that C4A/C4B in the brain and CSF was protective against SCZ. One explanation is that C4A and C4B were measured together in the pQTL, where the effect of C4A could be compromised by C4B. On the other hand, the pQTL was derived from the parietal lobe, while a previous study found that overexpression of C4A in the prefrontal cortex will lead to SCZ-associated phenotypes [29], which emphasized the tissue-specific role of C4. Together with these studies, our data suggest that the role of C4 in SCZ needs further exploration, and simply overexpressing the protein level of C4A/C4B might not be a promising therapeutic strategy for SCZ; more profound mechanism studies would be needed.

Moreover, we found that in the CSF, an increased abundance of TIE1, BCL6, and MICB was leading to a higher risk of SCZ, while an increased abundance of ACP5, CNTN2, and PLA2G7 was leading to a lower risk of SCZ. All of these genes have been rarely studied in SCZ, while there were some pieces of evidence supporting our results. For example, SNPs in *MICB* and *PLA2G7* (also known as PAFAH) have been found to be associated with SCZ risk [30–32]; CNTN2 could interact with Contactin-associated protein-like 2, which is a large multidomain neuronal adhesion molecule implicated in a number of neurological disorders, including epilepsy, schizophrenia, and autism spectrum disorder [33]. However, further investigation is needed to explore how these proteins involve in the pathogenesis of SCZ.

Furthermore, we found a robust positive correlation of the MR effects between plasma- and CSF-based proteins, while some proteins in the CSF and plasma also have a contrary role on SCZ, which might be due to the brain-blood barrier. Moreover, we did not find any correlation between CSF and the brain, which may be due to the limited number of proteins measured in the brain.

In the PPI network analysis, we found interactions between proteins from different tissues, and the proteins identified by MR were enriched in the biological process of “immune response” and “inflammatory response.” Therefore, our results further supported that immunity of both the peripheral and central nervous system (CNS) could work together to contribute to neuroinflammation, which is important in the pathogenesis of schizophrenia [34, 35].

There were some limitations in the current study. First, there were limited proteins measured in the brain, CSF, and plasma in the exposure GWAS [12], and most of the pQTLs had only 1 or 2 IVs available after selection, which made it not enforceable to perform sensitivity analyses and post-MR analyses; therefore, studies with larger sample sizes and more proteins would be needed. Second, the tissue types in our study were limited to the human parietal lobes, while some other brain regions were found to be more relevant to SCZ, such as the frontal cortex, hippocampus, amygdala, and para-hippocampus [36]. Therefore, genetic data derived from those regions will be needed to characterize candidate proteins. Thirdly, the pQTL datasets used in our study were obtained from AD cases and cognitively normal older adults, although the associations of the genetic variants with protein levels were found to be not disease-specific and not age-specific [12]; pQTLs from purely healthy controls with different ages would be better. Moreover, MR estimates represent the associations between life-long levels in the change of exposure with the outcome. However, most therapeutic interventions, particularly in clinical trials, are not life-long. Therefore, our MR results were likely to overestimate the therapeutic prospects of the identified proteins. Nonetheless, we believe it is reasonable that our MR results provided insights into studying the mechanism of SCZ. Last but not least, the GWAS datasets used in the current study originated from the European population, which may not reflect the conditions in other ethnicities because of the genetic heterogeneity; more studies from other races would be needed.

## Conclusions

In conclusion, we identified one protein in the brain and six proteins in the CSF that showed supporting evidence of being potentially causal for SCZ, which could provide insights into future mechanistic studies to find new treatments for the disease. Our results also supported the important role of neuroinflammation in the pathogenesis of SCZ.

## Supplementary Information

**Additional file 1: Table S1.** Cohort information. **Table S2.** Demographics of the participants included in the pQTL analyses. **Table S3.** IVs used in the MR analysis. **Table S4.** Phennoscanner results for the IVs. **Table S5.** MR results of proteins in the brain. **Table S6.** MR results of proteins in the CSF. **Table S7.** MR results of proteins in the plasma. **Table S8.** GO biological process enrichment for the suggestive proteins.

**Additional file 2: Figure S1.** 1A. Correlation of MR-effect between plasma and CSF (no p-value threshold); 1B. Correlation of MR-effect between plasma and brain (no p-value threshold); 1C. Correlation of MR-effect between the brain and CSF(no p-value threshold).

## Data Availability

The summary statistics of the pQTLs can be accessed via emailing the corresponding author in doi:10.1038/s41593-021-00886-6. GWAS for SCZ can be downloaded from the Psychiatric Genomics Consortium (https://pgc.unc.edu/).

## Abbreviations

SCZ: Schizophrenia
GWAS: Genome-wide association studies
MR: Mendelian randomization
SNP: Single nucleotide polymorphism
IV: Instrumental variable
pQTL: Protein quantitative trait loci
CSF: Cerebrospinal fluid
PPI: Protein-protein interaction
AD: Alzheimer’s disease
PGC: Psychiatric Genomics Consortium
LD: Linkage disequilibrium
IVW: Inverse-variance-weighted
PPH4: Posterior probability of Hypothesis 4
GO: Gene Ontology
STRING: Search Tool for the Retrieval of Interacting Genes
C4A/C4B: Complement 4A and 4B
IL36A: Interleukin 36A
TIE1: Tyrosine kinase with immuno-globulin-like and EGF-like domains 1
BCL6: BCL6 transcription repressor
MICB: MHC class I polypeptide-related sequence B
ACP5: Acid phosphatase 5
SERPING1: Protease inhibitor C1
CNTN2: Contactin 2
PLA2G7: Phospholipase A2 group VII
CTSS: Cathepsin S
IFNGR2: Interferon gamma receptor 2
eQTL: Expression quantitative trait loci
PWAS: Proteome-wide association analysis
TWAS: Transcriptome-wide association study
SMR: Summary-data-based Mendelian randomization
