# Supplementary figures and images for: Identifying novel proteins underlying schizophrenia via integrating pQTLs of the plasma, CSF, and brain with GWAS summary data
Source: BMC Med. 2022 Dec 8;20:474. doi: 10.1186/s12916-022-02679-5 (PMC9730613; doi:10.1186/s12916-022-02679-5)

## Additional file 2: Figure S1

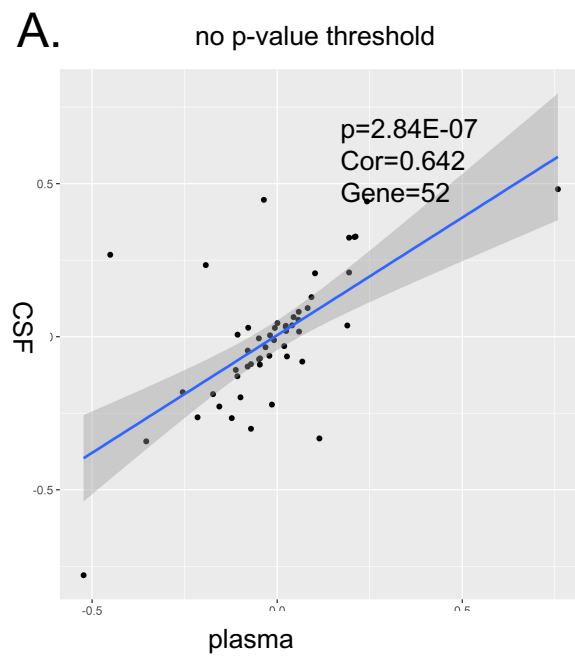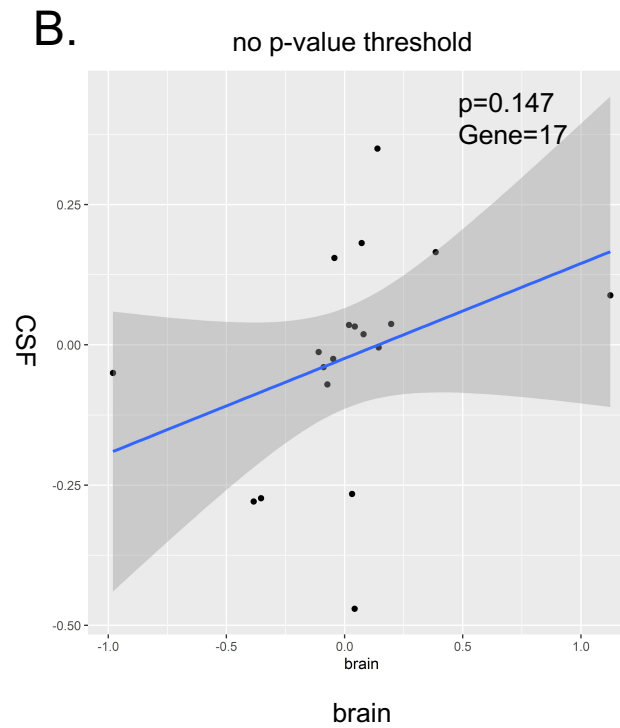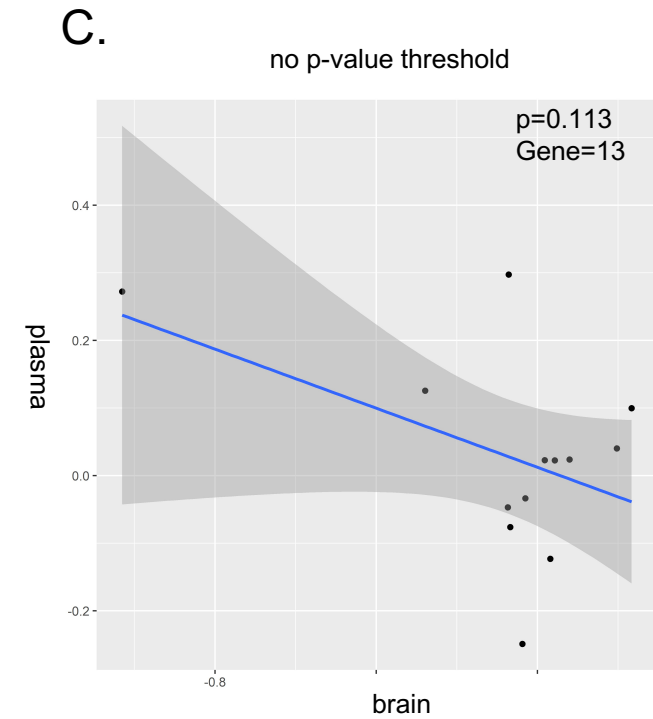

Supplement: Supplementary file 2 — Additional file 2: Figure S1. 1A. Correlation of MR-effect between plasma and CSF (no p-value threshold); 1B. Correlation of MR-effect between plasma and brain (no p-value threshold); 1C. Correlation of MR-effect between the brain and CSF(no p-value threshold). [file 12916_2022_2679_MOESM2_ESM.pdf]
